# Prognostic Value of Serum Free Light Chains Measurements in Multiple Myeloma Patients

**DOI:** 10.1371/journal.pone.0166841

**Published:** 2016-11-28

**Authors:** José Luis García de Veas Silva, Carmen Bermudo Guitarte, Paloma Menéndez Valladares, Johanna Carolina Rojas Noboa, Krysta Kestler, Rafael Duro Millán

**Affiliations:** 1 Department of Clinical Biochemistry, Hospital Universitario Virgen Macarena, Seville, Spain; 2 Department of Immunology, Complejo Hospitalario Universitario de Granada, Granada, Spain; 3 Department of Hematology, Hospital Universitario Virgen Macarena, Seville, Spain; Sudbury Regional Hospital, CANADA

## Abstract

**Background:**

The outcome for patients with Multiple Myeloma (MM) is highly variable, therefore, the existence of robust and easy to determine prognostic markers is extremely important for an efficient management of these patients. Presently, there is a debate about the role of the serum free light chains (sFLC) in the prognosis of MM patients both at diagnosis and after treatment. The aim of this study is to evaluate in a cohort of newly diagnosed MM patients from the Southern area of Spain, the prognostic value of sFLC both at baseline and after treatment.

**Materials and Methods:**

180 patients with a median age of 69 years were followed-up for a median time of 35 (18–61) months. The sFLC ratio (sFLCR) was calculated using the monoclonal sFLC as numerator. Patients were divided in two groups according to a sFLCR cut-off based on ROC analysis. The primary endpoints were the Overall Survival (OS) and the Progression-free Survival (PFS). Additionally, thirty-six MM patients treated with novel agents (Bortezomib/Dexamethasone) that achieved Complete Response (CR) or stringent CR (sCR) before autologous stem cell transplantation were studied to assess the impact of sCR in Disease Free Survival (DFS) and OS.

**Results:**

During follow-up there were 72 disease-related deaths. The 5-years OS for the whole group was 51%. However, separate analysis of patients with sFLCR above (group “high”) or below (groups “low”) the cut-off value of 47 shows an OS of 23% and 73%, respectively (HR = 5.03, 95%CI 2.99–8.50, p<0.001). In addition, analysis by ISS stage, showed that the presence of high sFLCR was always significantly associated with a worse OS. Multivariate analysis identified sFLCR (HR = 4.42, 95%CI 2.57–7.60, p<0.001) and beta-2-microglobulin (B2M) (HR = 3.04, 95%IC 1.75–5.31, p<0.001) as independent risk factors for adverse outcome. A new risk stratification model based on sFLCR≥47 and B2M>3.5 mg/L provided a statistically more significant result for this cohort when compared with the conventional ISS system. The HR for the new model were 2.84 (95% CI, 1.39–5.79, p = 0.004) for patients in stage 2 and 15.39 (95% CI, 6.35–37.33, p<0.001) for those in stage 3. Finally, in the group of patients reaching CR (19/36) or sCR (17/36) after induction, the median DFS for CR patients was 29 months, and NR for sCR patients (HR = 3.73; 95% CI 1.15–12.13, p = 0.03). Importantly, achieving sCR also translated into a significantly longer OS (5y-OS: sCR-89% versus CR-49%; p = 0.003; OS: sCR-NR versus CR-52 months).

**Conclusions:**

Our findings confirm the observations that the sFLCR has a major role in the survival of MM patients. A cut-off of sFLCR≥47 was shown to have an independent prognostic value at diagnosis, and a proposed “New Staging System” allows an accurate and simple method to risk stratify MM patients. Furthermore, because achievement of sCR was shown to represent a response state deeper than conventional CR resulting in greater OS and DFS, our study supports the continuity of sFLC ratio as part of the response criteria for MM patients.

## Introduction

Multiple Myeloma (MM) is a plasma cell dyscrasia characterized by the production of monoclonal immunoglobulins. The diagnostic and response criteria recommended have been defined by the International Myeloma Working Group (IMWG) [1,2]. The prognosis of patients with MM is highly variable due to the heterogeneity in biology of myeloma cells, bone marrow microenvironment and host factors. It is very important to identify risk groups of patients, as it will allow to optimize and begin the most appropriate treatment as quickly as possible, to avoid irreversible organ damage.

The International Staging System (ISS) is the current standard for staging of MM. The patients are categorized based on serum albumin and beta-2 microglobulin levels [3]. With the introduction of novel agents (Bortezomib, Lenalidomide, Thalidomide) the validity of ISS has been questioned [4,5]. The staging system was revised to include cytogenetics and lactate dehydrogenase [6]. The prognostic value of serum free light chains (sFLC) at diagnosis has been evidenced in monoclonal gammopathy of unknown significance, smouldering MM and solitary plasmacytoma [7–9]. Several recent studies have assessed the relationship between sFLC at disease presentation and prognosis of patients with MM [10–14].

In 2006, the IMWG introduced the evaluation of serum free light chains as part of the stringent Complete Response (sCR) category [15,16]. To date, there is discordance in the publications regarding the importance of this assessment, with large studies supporting the role of the assessment and others questioning the role of sFLC in the evaluation of response [17–20], clearly meaning that more work is need to definitively understand the role of sFLC in response. Intriguingly, Moustafa et al. reported recently that irrespective of the response achieved, the normalization of the sFLC ratio confers a better outcome [21].

The aim of this study is to evaluate the prognostic value of sFLC at baseline and to validate the role of sCR at remission status, in a cohort of newly diagnosed MM patients from the Southern area of Spain.

## Material and Methods

### Patients

A total of 180 patients with newly diagnosed MM (137 Intact Immunoglobulin MM (IIMM) and 43 Light Chain MM (LCMM)) were studied retrospectively during a period of seven years (from May 2008 to May 2015) at the Hospital Universitario Virgen Macarena (Seville, Spain). Patients were diagnosed by experienced Haematologists using the IMWG criteria [1]. Sixty-nine patients (39%) received treatment with novel agents based on bortezomib chemotherapy, 63 patients (35%) received treatment based on traditional agents and 31 patients (17%) received different lines of treatment. In 17 patients (9%) the treatment was not registered in the database. Clinical data of the patients including sex, age, myeloma subtype, disease stage based on ISS (3), haemoglobin, serum beta-2 microglobulin (B2M), serum calcium, serum creatinine, serum albumin, serum immunoglobulin, immunoparesis of non-monoclonal immunoglobulins, sFLC (kappa, lambda and ratio), serum lactate dehydrogenase (LDH), presence of lytic bone lesions, bone marrow (BM) plasma cell infiltration, serum protein electrophoresis (SPE), M-protein concentrations and immunofixation results were measured at the time of diagnosis. Quantitative variables were categorized as follow: haemoglobin<10 g/dL, calcium>11 mg/dL, creatinine>2 mg/dL, B2M>3.5 mg/L, albumin<3.5 g/dL, M-protein concentration>3 g/dL, BM plasma cell infiltration>20% and LDH>460 U/L. Haemoglobin was measured on a Sysmex 2000 autoanalizer; serum immunoglobulins, albumin, LDH, B2M, calcium, creatinine were measured on a COBAS 6000 autoanalizer. SPE were performed on CAPILLARYS 2^TM^ (Sebia) and the monoclonal component was identified by IFE on HYDRASYS^TM^ (SEBIA). The study was approved by the “Ethics Committee of Biomedical Research of Seville” with the code “MIELOMA”. Study based in data obtained from routine clinical practice, so no patient´s informed consent was given. All the data of the study were analysed anonymously.

### Serum free light chains assays

Serum free light chains were measured by turdibimetry using a latex-enhanced immunoassay (Freelite^TM^; The Binding Site Group Ltd, Birmingham, UK) on a SPA PLUS Specialist Protein Analyser. The involved *versus* uninvolved serum free light chain ratio (sFLCR) was calculated with the monoclonal light chain as numerator. For further survival analysis, patients were stratified in two groups according to the optimal sFLCR cut-off obtained by ROC curve analysis.

### Endpoints and response definitions

The primary endpoints of this study were overall survival (OS) and Progression-free survival (PFS). OS was defined as the time from initial diagnosis to death or the last follow-up and PFS was defined as time from initial diagnosis to date of progression, relapse or death. Patients who were lost to follow-up were censored at the date of last contact. A group of thirty-six patients with MM in treatment with novel agents (Bortezomib/Dexamethasone) that achieved Complete Response (CR) or sCR before autologous stem cell transplantation (ASCT) were studied for the impact of sCR in Disease Free Survival (DFS). DFS was defined as the time after treatment where disease remains stable. The definitions of CR and sCR were established using the IMWG criteria [15].

### Statistical analysis

Data cut-off was May 31, 2015. Pearson’s chi-squared test for categorical variables was used to compare patient’s characteristics among groups. Spearman´s correlation coefficients were calculated to study correlation between sFLC and clinical parameters. Receiver Operating Characteristic (ROC) curve analysis was performed to obtain the optimal cut-off of sFLCR in order to stratify patients in two groups for the survival analysis. OS, PFS and DFS were calculated by the method of Kaplan and Meier and the survival curves were compared using the Log-Rank test. Clinical and laboratory variables were evaluated for their impact on patient’s outcome. Variables found to be statistically significant were entered into a Cox regression proportional hazards model (stepwise regression method) to analyse the influence of prognostic factors in survival. Statistically significant factors were further tested in a logistic regression multivariate analysis with stepwise forward selection to evaluate prognostic ability of potential risk factors.

Discriminatory ability of each prognostic model was examined using the Cox proportional hazards model and the results expressed using the Akaike Information Criterion (AIC), a measure of relative goodness of fit of statistical models. A lower AIC indicates a more explanatory and informative model. A p value <0.05 was considered to be statistically significant for all comparisons. All p values are two-sided and confidence intervals refer to 95% boundaries. Statistical analyses were performed using Medcalc (version 16.8.4) and SPSS Statistics (version 18.0).

## Results

### Patient Characteristics

Baseline characteristics of the patients are shown in Table 1. Median age of the 180 patients included in the study was 69 years (range 61–76) and 54% were female. Abnormal sFLC ratio at baseline defined by K/L ratio <0.26 or >1.65 was observed in 98% of the patients. Median sFLC levels in patients with kappa light chain restriction (n = 95) was 254 mg/L (range 55–1053 mg/L) and 423 mg/L (range 156–1785 mg/L) in patients with lambda light chain restriction (n = 85). Median sFLCR for all patients was 50.79 (range 9.66–346.57). By ROC curve analysis, the optimal cut-off point for sFLCR with the highest sensitivity and specificity estimating survival was 46.95 (AUC = 0,634, p = 0,002) and, in terms of convenience, we rounded it to 47. Patients were stratified into two groups according this cut-off: low sFLCR with sFLC<47 (89 patients) and “high sFLCR” with sFLCR≥47 (91 patients).

**Table 1:** Characteristics of the MM patients at baseline and association with sFLCR (n = 180).

Parameters		All patients (n = 180)	sFLCR<47 (n = 89)	sFLCR≥47 (n = 91)	p value
**Male/Female (%)**		46/54	39/61	52/48	0.073
**Age, years (range)**		69 (61–76)	69 (63–76)	68 (59–76)	0.411
**Age>65 years**		116 (64)	61 (68)	55 (60)	0.562
**Intact Immunoglobulin MM**		137 (76)	73 (53)	64 (47)	0.128
	**IgG**	88 (64)	52 (59)	36 (41)	
	**IgA**	46 (34)	21 (23)	25 (54)	
	**IgD**	2 (2)	0 (0)	2 (100)	
	**IgM**	1 (1)	0 (0)	1 (100)	
**Light Chain MM**		43 (24)	17 (40)	26 (60)	0.05
	**Kappa**	20 (46)	11 (55)	9 (45)	
	**Lambda**	23 (54)	6 (26)	17 (64)	
**Creatinine>2 mg/dL**		32 (18)	10 (11)	22 (24)	0.023
**Haemoglobin<10 g/dL**		64 (36)	27 (42)	37 (58)	0.08
**Calcium>11 mg/dL**		17 (9)	6 (7)	11 (12)	0.220
**B2M>3,5 mg/L**		104 (58)	37 (42)	67 (74)	<0.001
**Albumin<3,5 g/dL**		61 (34)	27 (30)	34 (37)	0.319
**M-protein>3 g/dL**		72 (41)	27 (31)	45 (51)	0.007
**Immunoparesis**		133 (74)	55 (62)	78 (86)	<0.001
**LDH>460 U/L**		21 (12)	10 (11)	11 (12)	0.859
**Plasma cell infiltration % (range)**		20 (11–30)	14 (8–23)	24 (16–35)	<0.001
**Plasma cell infiltration>20%**		90 (50)	30 (34)	60 (66)	<0.001
**Presence of bone lesions**		117 (65)	49 (44)	68 (75)	0.006
**Plasmacytoma**		20 (11)	14 (16)	6 (6)	0.06
**ISS stage**					
	**ISS-1**	56 (31)	37 (42)	19 (21)	-
	**ISS-2 (vs. ISS-1)**	65 (36)	33 (37)	32 (35)	0.057
	**ISS-3 (vs. ISS-1)**	59 (33)	19 (21)	40 (44)	<0.001

Qualitative data expressed as n(%). Quantitative data expressed as median (interquartile range). B2M, beta-2-microglobulin; ISS, International Staging System; LDH, lactate dehydrogenase; MM, Multiple Myeloma; sFLCR, serum free light chains ratio

According to ISS, 31% patients had stage 1, 36% had stage 2 and 33% had stage 3. Forty-three patients were diagnosed of LCMM (46% with kappa restriction and 54% with lambda restriction) and 137 patients had IIMM. In the IIMM, IgG subtype was present in 64%, IgA in 34%, IgD in 2% and, finally, IgM in 1% of the patients. At diagnosis, the proportion of patients with renal impairment (creatinine>2 mg/dL), anemia (Hb<10 g/dL), hypercalcemia (Ca>11 mg/dL) and presence of bone lesions were 18%, 36%, 9% and 65%, respectively. The median percentage of BM plasma cells was 20% and 11% of patients had plasmacytoma at diagnosis. Immunoparesis of non-monoclonal immunoglobulin was observed on 133 patients (74%) and elevated serum LDH was noted in 12% of patients.

### Association of sFLC with parameters of disease activity

High sFLCR (sFLCR≥47) was associated with renal impairment (creatinine>2 mg/dL, p = 0.023), B2M>3.5 mg/L (p<0.001), M-protein concentration>3 g/dL (p = 0.007), immunoparesis (p<0.001), plasma cell infiltration>20% (p<0.001) and presence of lytic bone lesions (p = 0.006). No correlations were found with anemia (Hb<10 g/dL), hypercalcemia (Ca>11 mg/dL), albumin<3.5 g/dL and LDH>460 U/L (Table 1). There was a greater percentage of patients in ISS stage 3 in the sFLCR≥47 group (p<0.001).

Moreover, baseline sFLCR correlated with B2M, haemoglobin, creatinine and percentage of plasma cell infiltration in bone marrow (Table 2). No correlation was found with albumin, calcium, M-protein concentration and LDH levels in serum. Also, the correlation of sFLC levels with clinical parameters was evaluated in kappa and lambda restricted MM, separately. Regardless of the light chain isotype, sFLC levels showed a significant positive correlation with B2M, creatinine and percentage of plasma cell infiltration. Additionally, a significant negative correlation was observed between sFLC kappa levels and haemoglobin only in patients with kappa restricted MM. No correlation was found with the other clinical parameters (Table 2). Surprisingly, in disagreement with the observations from Kyrtsonis [10] and Xu [14], no correlation was found between sFLC levels and LDH.

**Table 2:** Correlation analysis between clinical parameters of the disease and sFLCR, sFLC kappa and sFLC lambda levels.

Clinical parameter of disease	sFLCR in all MM patients (n = 180)		sFLC kappa levels in kappa restricted MM (n = 95)		sFLC lambda levels in lambda restricted MM (n = 85)	
	Coefficient	p value	Coefficient	p value	Coefficient	p value
B2M	0.417	<0.001	0.414	<0.001	0.404	<0.001
Calcium	0.152	0.420	0.042	0.688	-0.036	0.744
Haemoglobin	-0.251	0.001	-0.314	0.002	-0.116	0.292
Creatinine	0.202	0.006	0.362	<0.001	0.268	0.013
Plasma cell infiltration	0.350	<0.001	0.225	0.028	0.386	<0.001
Albumin	-1.123	0.099	-0.082	0.430	-0.073	0.508
M-protein concentration	0.166	0.053	0.091	0.440	0.098	0.447
LDH	-0.014	0.848	-0.046	0.659	0.124	0.260

sFLCR; serum free light chains ratio, sFLC, serum free light chains; B2M, beta-2-microglobulin; LDH, lactate dehydrogenase, MM, Multiple Myeloma.

### High sFLCR associates with shorter survival

**Fig 1:**
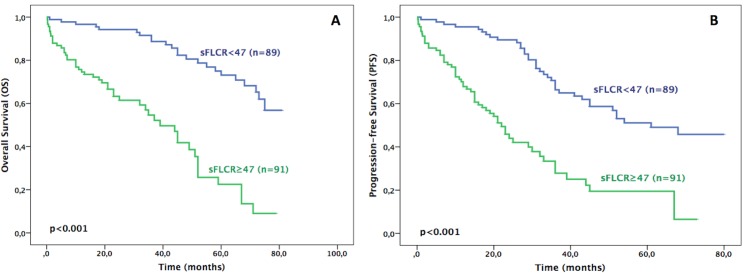
Prognostic value of sFLCR. (A) Overall Survival (OS) and (B) Progression-free survival (PFS) of all MM patients (n = 180) stratified according to a low (<47) or high (≥47) sFLCR.

The median follow-up was 35 months (range 18–61 months). During the study period, there were seventy-two disease-related deaths: forty-nine in the group of patients with sFLCR≥47 and 23 with sFLCR<47. The 5-years OS of all patients was 51%, whereas it was 73% and 23% in low and high sFLCR groups, respectively (HR = 5.03, 95% CI: 2.99–8.50, p<0.001). The median OS was not reached (NR) versus 39 months (95% CI: 29–48 months, p<0.001) for the low and high sFLCR groups, respectively (Fig 1A).

Furthermore, sFLC levels above the median values (kappa≥254 mg/L and lambda≥423 mg/L for kappa and lambda patients, respectively) were found to predict a worse prognosis. The 5-years OS was 63% for patients with sFLC levels below the median and 37% in those with sFLC levels above the median (HR = 2.44 95% CI: 1.50–3.97, p = 0.001). The median OS was NR versus 51 months (95% CI: 45–56 months, p = 0.001), respectively.

By univariate analysis, the other variables significantly associated with adverse outcome were: age>65 years old, creatinine>2 mg/dL, hemoglobin<10 g/dL, B2M>3.5 mg/L, albumin<3.5 g/dL, immunoparesis of non-monoclonal immunoglobulins and ISS stages 2 & 3. There was no significant correlation with the others variables. Multivariate Cox stepwise regression analysis of factors affecting OS identified sFLCR (HR = 4.42, 95% IC 2.57–7.60, p<0.001) and B2M (HR = 3.04, 95% IC 1.75–5.31, p<0.001) as independent risk factors for adverse outcome (Table 3).

**Table 3:** Univariate and multivariate analysis of prognostic factors for Overall Survival (OS) and Progression-free Survival (PFS) in patients with newly diagnosed MM (n = 180).

VARIABLES	OVERALL SURVIVAL (OS)				PROGRESSION-FREE SURVIVAL (PFS)			
	Univariate analysis		Multivariate analysis		Univariate analysis		Multivariate analysis	
	HR	p-value	HR	p-value	HR	p-value	HR	p-value
	(95% CI)		(95% CI)		(95% CI)		(95% CI)	
**Age>65 years**	1.99 (1.17–3.41)	0.01	-		1.66 (1.06–2.59)	0.03	-	
**Female sex**	0.81 (0.51–1.29)	0.3	-		0.92 (0.61–1.39)	0.6	-	
**sFLCR≥47**	5.03 (2.99–8.50)	<0.001	4.42 (2.57–7.60)	<0.001	3.71 (2.34–5.56)	<0.001	3.19 (2.05–4.96)	<0.001
**High sFLC levels (kappa≥254 mg/L and lambda≥423 mg/L)**	2.44 (1.50–3.97)	0.001	-		2.50 (1.64–3.81)	0.001	-	
**Creatinine >2 mg/dL**	2.15 (1.25–3.70)	0.005	-		2.61 (1.64–4.14)	0.001	-	
**Hemoglobin <10 g/dL**	1.95 (1.22–3.12)	0.005	-		1.50 (0.99–2.28)	0.06	-	
**Calcium >11 mg/dL**	1.75 (0.87–3.53)	0.2	-		1.69 (0.90–3.19)	0.1	-	
**B2M >3.5 mg/L**	3.66 (2.16–6.21)	<0.001	3.04 (1.75–5.31)	<0.001	2.58 (1.66–4.01)	<0.001	2.13 (1.35–3.35)	0.001
**Albumin <3.5 g/dL**	1.84 (1.14–2.96)	0.01	-		1.16 (0.76–1.79)	0.48	-	
**M-protein>3 g/dL**	1.86 (1.07–3.21)	0.03	-		1.01 (0.67–1.56)	0.93	-	
**Immunoparesis of non-monoclonal immunoglobulin**	1.74 (1.01–2.98)	0.04	-		1.73 (1.08–2.76)	0.02	-	
**LDH >460 U/L**	1.41 (0.74–2.68)	0.2	-		1.48 (0.82–2.66)	0.2	-	
**Plasma cell>20%**	1.34 (0.85–2.17)	0.2	-		1.48 (0.82–2.66)	0.2	-	
**Lytic bone lesions**	1.21 (0.74–1.97)	0.4	-		1.25 (0.82–1.91)	0.3	-	
**Plasmacytoma**	0.66 (0.28–1.51)	0.3	-		0.71 (0.34–1.47)	0.3	-	
**ISS-2 (vs. ISS-1)**	3.02 (1.56–5.73)	0.001	-		1.69 (1.08–2.83)	0.04	-	
**ISS-3 (vs. ISS-1)**	4.88 (2.50–9,52)	<0.001	-		3.09 (1.81–5.28)	<0.001	-	

B2M, beta-2-microglobulin; CI, confidence interval; ISS, International Staging System; LDH, lactate dehydrogenase; MM, Multiple Myeloma; OS, Overall Survival; PFS, Progression-Free Survival; sFLC, serum free light chains; sFLCR, serum free light chains ratio.

Regarding the PFS, during the study the 5-years PFS of all patients (n = 180) was 37% with a median PFS of 36 months (95% CI:27,44 months). Stratifying by sFLCR≥47, the 5-years PFS was 51% and 19% in low and high sFLCR groups; respectively (HR = 3.71 95% CI: 2.34–5.56, p<0.001). The median PFS was 61 months (95% CI: NR) versus 22 months (95% CI: 17–27 months, p<0.001) for the low and high sFLCR groups, respectively (Fig 1B). Again, by multivariate Cox stepwise regression analysis only sFLCR (HR = 3.19, 95% CI: 2.05–4.96, p<0.001) and B2M (HR = 2.13, 95% CI: 1.35–3.35, p = 0.001) remained statistically significant prognostic factors (Table 3).

Previously, different sFLC K/L ratio cut-offs were associated with prognosis in newly diagnosed MM patients. Table 4 assesses and compares the prognostic value of sFLC cut-offs obtained by Snozek (sFLC K/L ratio 0.03–32 or <0.03 >32) and Xu (sFLC K/L ratio 0.04–25 or <0.04 >25) with the sFLCR involved/uninvolved < or ≥47 obtained in this report. The different cut-offs performed well in terms of prognostication of survival, however we found that the sFLCR involved/uninvolved < or ≥47 had more prognostic power than the others cut-offs assayed.

**Table 4:** Prognostic value obtained with different sFLC cut-offs.

Authors	Cut-offs	N	PF (%)	HR (95% CI)	OS (%)	HR (95% CI)
Snozek [11]	0.03–32	75	54	3.76 (2.38–5.92), p<0.001	72	3.80 (2.23–6.45), p<0.001
	<0.03 or >32	105	21		32	
Xu [14]	0.04–25	70	55	3.67 (2.31–5.84), p<0.001	74	4.04 (2.33–6.99), p<0.001
	<0.04 or >25	110	23		32	
Garcia de Veas Silva (current report)	<47	89	51	3.71 (2.34–5.56), p<0.001	73	5.03 (2.99–8.50), p<0.001
	≥47	91	19		23	

CI, Confidence Interval; HR, Hazard Ratio; OS, Overall Survival; PFS, Progression-Free Survival

### sFLCR has prognostic value irrespective of the treatment

**Fig 2:**
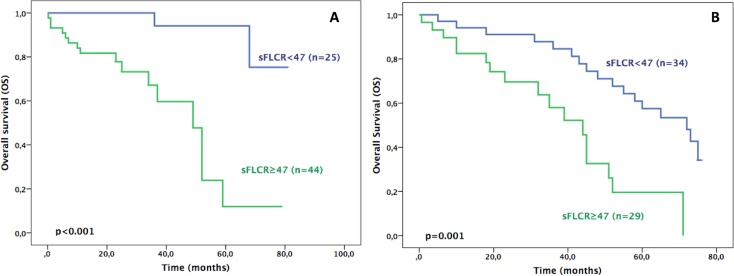
Prognostic value of sFLCR regarding the treatment scheme (A) Overall Survival (OS) of patients under treatment with novel agents (n = 69) stratified by sFLCR. (B) Overall Survival (OS) of patients under treatment with traditional agents (n = 63) stratified by sFLCR.

Next, patients were divided in two groups based on the treatment received: the traditional agents group (n = 63) and the novel agents group (n = 69). The median OS for patients in novel agents group and traditional agents group were 68 and 52 months, respectively. The further stratification of these two groups by sFLCR levels allowed us to study the prognostic value of sFLCR in each treatment group. In the novel agents group of patients, the 5-years OS in high (sFLCR≥47) and low (sFLCR<47) groups were 12% and 94%, respectively (p<0.001). The high sFLCR group showed a median OS of 49 months (95% CI: 37–60 months) versus NR by the low sFLCR group (Fig 2A). Similarly, in the traditional agents group of patients, the 5-years OS was 19% in high sFLCR group and 57% in low sFLCR group, with a median OS of 44 months (95% CI: 35–52 months) and 72 months (95% CI: 55–88 months), respectively (p = 0.001) (Fig 2B).

### Stratification of patients by sFLCR in combination with ISS

**Fig 3:**
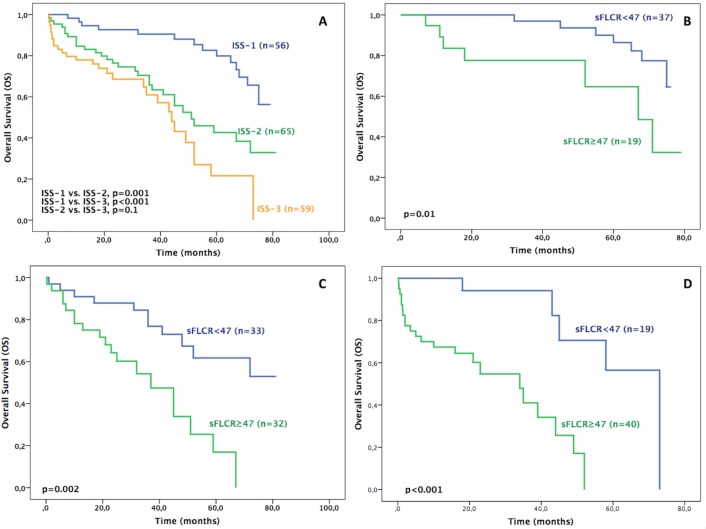
A) Overall Survival (OS) of all patients based on International Staging System (ISS) (n = 180). B) OS of patients in ISS-1 stratified by sFLCR (n = 56). C) OS of patients in ISS-2 stratified by sFLCR (n = 65). D) OS of patients in ISS-3 stratified by sFLCR (n = 59).

In our cohort of patients, according to ISS stages 1, 2 and 3, the median OS of all patients were NR, 51 and 44 months, respectively. However, the difference found between stages 2 and 3 was not statistically different (p = 0.1) (Table 5 and Fig 3A). The ISS was combined with the presence of high or low sFLCR to check its prognostic value within every ISS stage. The sFLCR separated every ISS stages in two subsets: one with low sFLCR (sFLCR<47) and another with high sFLCR (sFLCR≥47). Within every ISS stage, the presence of high sFLCR was associated with a worse OS (Table 5 and Fig 3B–3D). The HR associated with a high sFLCR were: 3.82 (95% CI, 1.32–11.10, p = 0.01), 3.58 (95% CI, 1.62–7.90, p = 0.002) and 7.91 (95% CI, 2.32–26.91, p = 0.001) for stages 1, 2 and 3, respectively.

**Table 5:** Prognostic value of International Staging System (ISS) and stratified by sFLCR.

**Risk groups**	**N**	**Number of deaths n (%)**	**5-years OS (%)**	**Median OS (months)**	**HR (95% CI)**	**p-value**
**ISS-1**	**56**	**14 (25)**	**79**	**NR**	**1.00 (reference)**	**-**
**sFLCR<47**	37	7 (19)	86	NR	1.00 (reference)	-
**sFLCR≥47**	19	7 (36)	65	67 (46–87)	3.82 (1.32–11.10)	0.01 ^(1)^
**ISS-2**	**65**	**30 (46)**	**43**	**51 (34–67)**	**3.02 (1.59–5.73)**	**0.001** ^**(2)**^
**sFLCR<47**	33	11 (33)	62	NR	1.00 (reference)	-
**sFLCR≥47**	32	19 (59)	17	37 (20–53)	3.58 (1.62–7.90)	0.002 ^(1)^
**ISS-3**	**59**	**28 (47)**	**22**	**44 (36–51)**	**4.88 (2.50–9.52)**	**<0.001** ^**(3)**^**, 0.1** ^**(4)**^
**sFLCR<47**	19	5 (26)	56	73 (NR-NR)	1.00 (reference)	-
**sFLCR≥47**	40	23 (58)	0	34 (19–48)	7.91 (2.32–26.91)	<0.001 ^(1)^
**Risk groups**	**N**	**Number of events n (%)**	**5-years PFS (%)**	**Median PFS (months)**	**HR (95% CI)**	**p-value**
**ISS-1**	**56**	**25 (45)**	**54**	**67 (41–92)**	**1.00 (reference)**	**-**
**sFLCR<47**	37	16 (43)	58	68 (NR-NR)	1.00 (reference)	-
**sFLCR≥47**	19	9 (47)	47	36 (8–63)	2.25 (1.01–5.19)	0.04 ^(1)^
**ISS-2**	**65**	**35 (54)**	**34**	**45 (30–59)**	**3.02 (1.59–5.73)**	**0.04** ^**(2)**^
**sFLCR<47**	33	14 (42)	44	52 (50–53)	1.00 (reference)	-
**sFLCR≥47**	32	21 (65)	22	23 (13–32)	2.80 (1.39–5.65)	0.003 ^(1)^
**ISS-3**	**59**	**35 (59)**	**15**	**27 (21–32)**	**4.88 (2.50–9.52)**	**<0.001** ^**(3)**^**, 0.02** ^**(4)**^
**sFLCR<47**	19	7 (36)	41	36 (26–46)	1.00 (reference)	-
**sFLCR≥47**	40	28 (70)	0	19 (13–25)	4.95 (2.01–11.69)	<0.001 ^(1)^

^(1)^ p-value within groups defined by sFLCR<47 and sFLCR≥47.

^(2)^ p-value between ISS-1 and ISS-2 stages.

^(3)^ p-value between ISS-1 and ISS-3 stages.

^(4)^ p-value between ISS-2 and ISS-3 stages.

CI, confidence interval; ISS, International Staging System; NR, not reached; OS, Overall Survival; PFS, Progression-Free Survival; sFLCR, serum free light chains ratio.

**Fig 4:**
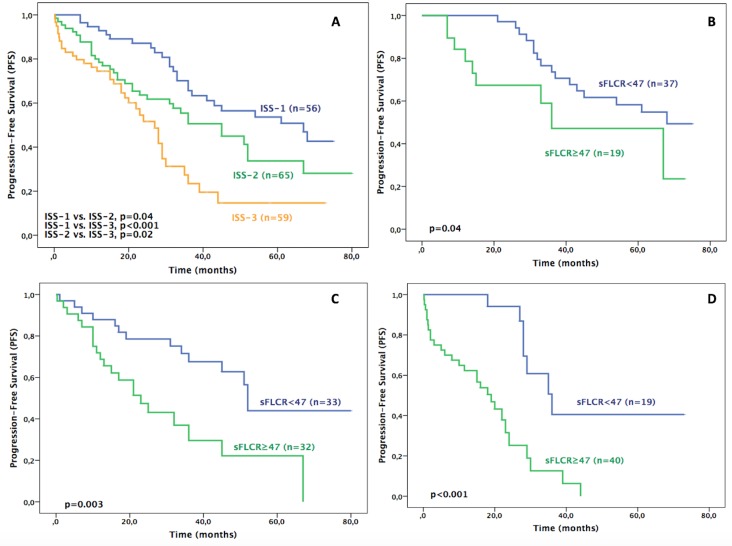
A) Progression-free survival (PFS) of all patients based on International Staging System (ISS) (n = 180). B) PFS of patients in ISS-1 stratified by sFLCR (n = 56). C) PFS of patients in ISS-2 stratified by sFLCR (n = 65). D) PFS of patients in ISS-3 stratified by sFLCR (n = 59).

PFS median values were 67, 45 and 27 months for ISS stages 1,2 and 3, respectively. Similarly to OS, the presence of high sFLCR was associated with worse PFS (Table 5 and Fig 4A–4D). The HR associated with a high sFLCR were: 2.25 (95% CI, 1.01–5.19, p = 0.04), 2.80 (95% CI, 1.39–5.65, p = 0.003) and 4.95 (95% CI, 2.01–11.69, p<0.001) for stages 1, 2 and 3, respectively.

### Alternative staging system based on sFLCR and B2M

**Fig 5:**
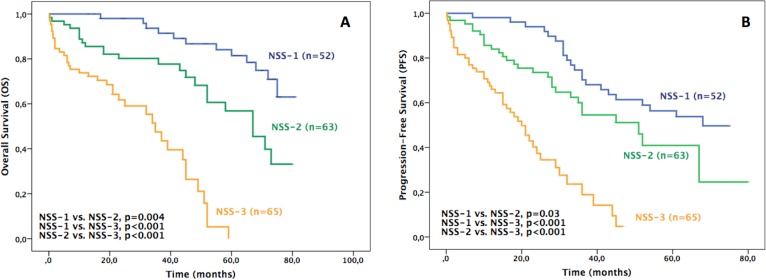
(A) Overall Survival (OS) and (B) Progression-Free Survival (PFS) of all patients based on New Staging System (NSS) (n = 180).

According to the Cox Regression multivariate analysis, in this series of patients the sFLCR and B2M showed to be the independent adverse prognostic factors (Table 3). Based on these two parameters, an alternative staging system denominated “New Staging System (NSS)” was created using their respective cut-offs defined in this study. Thereby, this new model has three stages defined as follows: stage 1 (B2M<3.5 mg/L and sFLCR<47), stage 2 (B2M>3.5 mg/L or sFLCR≥47) and stage 3 (B2M>3.5 mg/L and sFLCR≥47). The median OS obtained with this “New Staging System” were NR, 67, and 35 months for stages 1, 2 and 3, with a 5-years OS of 81%, 56% and 0%, respectively. Importantly, statistically significant differences were found between stages 2 and 3 (p<0.001) (Table 6 and Fig 5A). The associated HR was 2.84 (95% CI, 1.39–5.79, p = 0.004) for patients in stage 2 and 15.39 (95% CI, 6.35–37.33, p<0.001) for those in stage 3.

**Table 6:** Prognostic value of “New Staging System (NSS)” based on sFLCR and B2M for Overall Survival (OS) and Progression-Free Survival (PFS).

**Risk groups**	**N**	**Number of deaths n (%)**	**5-years OS (%)**	**Median OS (months)**	**HR (95% CI)**	**p-value**
**NSS-1 (B2M<3.5 mg/L and sFLCR<47)**	52	12 (23)	81	NR	1.00 (reference)	-
**NSS-2 (B2M>3.5 mg/L or sFLCR≥47)**	63	23 (37)	56	67 (52–81)	2.84 (1.39–5.79)	0.004 ^(1)^
**NSS-3 (B2M>3.5 mg/L and sFLCR≥47)**	65	37 (57)	0	35 (28–41)	15.39 (6.35–37.33)	<0.001 ^(2)^, <0.001 ^(3)^
**Risk groups**	**N**	**Number of events n (%)**	**5-years PFS (%)**	**Median PFS (months)**	**HR (95% CI)**	**p-value**
**NSS-1 (B2M<3.5 mg/L and sFLCR<47)**	52	22 (42)	56	68 (NR-NR)	1.00 (reference)	-
**NSS-2 (B2M>3.5 mg/L or sFLCR≥47)**	63	29 (46)	41	51 (36–65)	1.83 (1.04–3.22)	0.03 ^(1)^
**NSS-3 (B2M>3.5 mg/L and sFLCR≥47)**	65	44 (68)	0	20 (15–25)	6.02 (3.41–10.63)	<0.001 ^(2)^, <0.001 ^(3)^

^(1)^ p-value between stages 1 and 2.

^(2)^ p-value between stages 1 and 3.

^(3)^ p-value between stages 2 and 3.

B2M, beta-2-microglobulin; CI, confidence interval; NR, not reached; NSS, New Staging System; OS, Overall Survival; PFS, Progression-Free Survival; sFLCR, serum free light chains ratio

The median PFS obtained with the “New Staging System” were 68, 51, and 20 months for stages 1, 2 and 3 with a 5-years PFS of 56%, 41% and 0%, respectively. The risk of progression was increased for patients in stage 2 (HR = 1.83, 95% CI, 1.04–3.22, p = 0.03) and for patients in stage 3 (HR = 6.02, 95% CI, 3.41–10.63, p<0.001) (Table 6 and Fig 5B).

### Comparison of prognostic models

Based of the results obtained for ISS and NSS models, the prognostic ability for both models for OS and PFS using the AIC values were compared (Table 7). According to the values obtained, the NSS showed the lowest AIC value for OS and PFS being identified as the most accurate staging system in stratifying this cohort of patients in comparison with ISS.

**Table 7:** Comparison of the prognostic ability of the International Staging System (ISS) and New Staging System (NSS) using the Akaike Information Criterion (AIC) values.

Prognostic models	AIC values for OS	AIC values for PFS
**International Staging System (ISS)**	634.896	857.835
**New Staging System (NSS)**	607.627	831.835

AIC, Akaike Information Criterion; OS, Overall Survival; PFS, Progression-free Survival

### Impact of stringent CR in patients treated with novel agents

**Fig 6:**
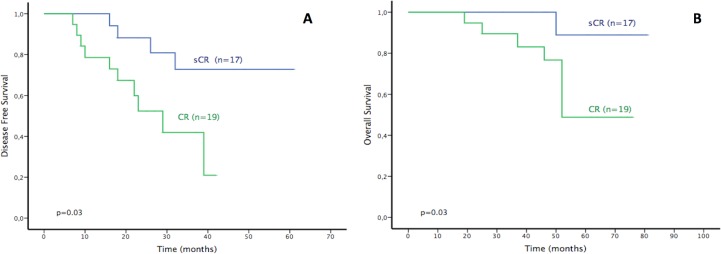
(A) Disease Free Survival (DFS) of the patients achieving Complete Response (CR) (n = 19) and stringent CR (sCR) (n = 17). (B) Overall Survival (OS) of the patients achieving Complete Response (CR) (n = 19) and stringent CR (sCR) (n = 17).

Finally, the impact of sCR as prognostic factor of remission was evaluated in thirty-six patients that achieved sCR or CR after treatment with Bortezomib/Dexamethasone. Nineteen (52.8%) patients achieved CR and seventeen (47.2%) patients achieved sCR. During the study period, there were fourteen relapses, ten in patients achieving CR and four in patients achieving sCR (Fig 6A). The median DFS for patients achieving CR was 29 months (95% CI: 18–39 months) and NR for those achieving sCR. Patients achieving CR had a DFS rate of 21% compared with 73% for sCR (HR = 3.73; 95% CI 1.15–12.13, p = 0.03).

Furthermore, sCR was associated with longer OS in comparison to CR. Of the thirty-six patients studied, there were nine disease-related deaths during the study: one in the sCR group and eight in the CR group. The 5-years OS was 89% in patients achieving sCR compared to 49% in those in CR (p = 0.03). The median OS was NR for patients in sCR versus 52 months for those in CR (Fig 6B).

## Discussion

This study investigated the prognostic value of sFLC in a cohort of Spanish patients with newly diagnosed MM, and the validation of sCR in a subgroup of these patients. In concordance with previous studies, 98% of patients presented with an abnormal serum free light chains ratio (against a published reference range of 0.256–1.65) [11,22]. High sFLCR (sFLCR≥47) is associated with creatinine, B2M, M-protein concentration, immunoparesis, plasma cell infiltration>20%, presence of lytic bone lesions and ISS stage 3 (Table 1). The association with these parameters support the prognostic value of sFLC because they are indicative of higher tumour burden, deterioration of renal function and a more aggressive disease. Similar findings have been found previously [12,13]. Furthermore, a positive correlation was confirmed between sFLCR, free kappa and free lambda with plasma cell infiltration in bone marrow, creatinine and B2M (Table 2).

The relationship between sFLC at diagnosis and disease outcome have been assessed in previous studies. Kyrtsonis et al. [10] showed for the first time that high sFLC ratio, defined as involved/uninvolved, highly correlated with survival independently of the ISS. The 5-year disease specific survival was 82% and 30%, respectively for patients with involved/uninvolved sFLC ratios < or ≥ the median values. Similarly, in a study at the Mayo Clinic [11], an abnormal sFLC ratio at diagnosis was an independent marker of prognosis. They concluded that those patients with a sFLC ratio <0.03 or >32 presented a worse prognosis with median survival of 30 months compared with 48 months for patients with ratios within the normal range. The authors suggested that sFLC ratio could be incorporated into ISS to improve the risk stratification of patients with MM.

In keeping with previous studies, patients with sFLC ratio above the cut-off (sFLCR≥47) had a poorer overall survival. However, these studies lack consensus with respect to the cut off [11,13,14]. The results from this study confirmed the prognostic value of sFLCR in a cohort of patients in the southern area of Spain for the first time, with a cut-off sFLCR≥47 predicting a worse outcome. The sFLCR cut-off obtained in this study is higher than reported by Snozek et al. (sFLC ratio <0.03 or >32) [11] and by Xu et al. (sFLC ratio <0.04 or >25) [14], but it is close to that obtained by Sthaneshwar et al. with median sFLC ratios of >57.5 and 0.04 for kappa and lambda secretors MM [13].

Using the three cut-offs (Snozek, Xu and García de Veas Silva) significant PFS and OS differences were found in out cohort of patients (Table 4). While for PFS the HR were similar for the three cut-offs, in relation to OS, our cut-off provided a higher HR. Therefore, the sFLCR≥47 cut-off was selected to be included in the NSS instead of the others two cut-offs.

According to ISS, in the present study, the median OS of patients in stages 1, 2 and 3 were NR, 51 and 44 months, respectively. Nevertheless, the ISS could not discriminate the outcome of patients in stages 2 and 3 (p = 0.1). Combining sFLC with ISS allowed to divide patients in two groups with different prognosis within each ISS stage. Consistent with the previous report by Kryrtsonis et al. [10], these results confirm the association between sFLCR and prognosis in every ISS stage. Nonetheless, although this finding was also observed by Snozek et al. [11] in patients in ISS stage 2 but not in stages 1 and 3, they suggested the inclusion of baseline sFLC ratio into the ISS and the need to validate it in multicentric studies.

Although the ISS is the widely accepted prognostic staging system for patients with MM, the validity of this system has been questioned in the era of the novel therapeutic agents [4,5,23]. Iriuchishima et al. showed that OS of patients treated with conventional chemotherapy is significantly different depending on stages of ISS but this staging system has no effect on OS in patients under therapy with novel agents [5]. The presence of a sFLCR<47 was associated with a longer OS both in patients under treatment with traditional agents and novel agents, separately. In fact, the effect of stratifying by sFLCR was more significant in patients treated with novel agents (median OS NR versus 49 months for patients with sFLCR<47 and ≥47, respectively, p<0.001) than those with traditional agents (median OS of 72 and 44 months, respectively, p = 0.001). This result is in agreement with the described by Xu et al., where a high sFLC ratio was associated with shorter median OS, regardless of the treatment including novel or traditional agents [14].

The cytogenetic abnormalities, included in the revised ISS, are considered one of the most important prognostic factors in patients with MM. Because the cytogenetic abnormalities were not recorded for most of the patients included, cytogenetics abnormalities or the revised ISS could not be evaluated on this study. Nevertheless, some studies have evidenced that cytogenetic abnormalities were associated with high sFLC levels and sFLC ratio. Kumar et al. [24] demonstrated that higher sFLC levels and abnormal ratios are frequently detected in patients with IgH translocations and are associated with poor prognosis. Interestingly, t(14;16) was especially associated with higher sFLC levels and abnormal sFLC ratio. In another study [25], both high sFLC ratio and sFLC difference were associated with abnormal karyotypes, complex karyotypes and high plasma cell burden. The associations between the cytogenetic abnormalities and sFLC found in these studies supports our finding that sFLCR is a powerful prognostic factor in patients with MM. Furthermore, the sFLC present advantages over the cytogenetic abnormalities such as that they are inexpensive, quickly and easily to determine in a blood serum sample.

In the current study, only sFLCR and B2M remained significant prognostic factors for OS and PFS on a multivariate analysis. Noteworthy, although it has been described that LDH has prognostic impact on survival [4,25], we found no relationship between LDH and survival in our series of patients. Therefore, we propose a risk stratification model based on the two parameters, sFLCR and B2M. The results indicate that the NSS based on sFLCR and B2M is a better prognostic model for MM patients in terms of OS and PFS compared with ISS. These findings were confirmed by the AIC analysis (Table 7) that provides information about the predictive accuracy of prognostic models. The AIC analysis represents an overall assessment of prognostic system being an important reference for the comparison between different staging systems. The NSS showed the lowest AIC value in comparison with ISS. This suggest that the prognostic power of NSS is homogeneously stable across different risk groups. Furthermore, it is important for planning treatment strategies in MM patients based on the prediction of prognosis and improving the design of clinical trials.

At response, the achievement of CR is an independent predictor of PFS and OS in patients under treatment with novel agents [26]. The sCR, a new category defined by the IMWG, requires achieving a CR plus normalization of sFLC ratio and absence of clonal plasma cells by immunohistochemistry or 2 to 4-color flow cytometry [15,16].

Recently, Kapoor et al. [18] have shown that patients who achieve a sCR have a better outcome compared to those with a lower depth of response treatment. Patients with a sCR after ASCT showed a superior OS and longer time to progression (TTP) than those who achieved a CR. The median TTP of patients achieving sCR was significantly longer than those in CR (50 months versus 20 months, respectively) and the patients in sCR presented a marked improvement in OS compared to patients in CR (NR versus 81 months, respectively). On the other hand, Radocha et al. [19] did not observed better outcome in terms of PFS and OS in myeloma patients achieving sCR. Nevertheless, the induction regimens before transplantation in that study varied which could produce a significant bias in the validation of sCR. Paiva et al. [20] observed that achieving a immunophenotyping response (IR) was associated with superior PFS and TTP compared with CR and sCR without differences in survival between CR and sCR. Nonetheless, sCR was defined a CR plus normal sFLC ratio not as defined as IMWG.

Importantly, our results expand the degree of validity observed recently by Kapoor et al. [18]. The DFS of patients achieving sCR was significantly superior to those in CR with median values of NR and 29 months, respectively. Furthermore, the OS was higher in the patients achieving sCR compared to those in CR with 5-years OS of 79% and 49%, respectively. This study has the limitation of a small sample size of patients. However, we consider an advantage that it considers a homogeneous group of patients with induction therapy based only in novel agents (Bortezomib/Dexamethasone) that achieved CR or sCR and underwent ASCT. Patients under treatment with conventional agents were not included. We consider that achieving sCR could have a major role in the treatment of patients with novel agents and more clinical studies with larger series of patients are needed to validate the category of sCR.

A study by Iwama et al. in a population of 126 patients with MM showed the favourable effect of sFLC ratio normalization on the survival and suggests the importance of sFLC in the prognosis of these patients. A normal sFLC ratio was associated with longer OS and PFS, the positive effect of a normal sFLC ratio on OS was observed among different IMWG response groups. Patients with normal sFLC ratio showed a better OS compared to those without sFLC ratio normalization among groups of patients with CR, very good partial response (VGPR) and partial response (PR) [17]. More recently, the results of Moustafa et al. [21] support the inclusion of sFLC in all levels of response criteria defined by the IMWG. They showed that the impact of the normalization of sFLC provides a more favourable prognosis irrespective of the degree of response in patients who did not achieve a CR.

This study has some limitations. First, the sample size was small, particularly in the validation of sCR in patients treated with novel agents. Second, the data from patients achieving other degrees of response could not be collected to evaluate the prognostic value of sFLC in other response categories, as it was reported by Moustafa et al. [21]. However, the NSS model incorporating sFLCR and B2M could serve as an objective and accurate prognostic model in patients with MM. The high statistical significance between stages in NSS, especially among stages 2 and 3, besides the lowest AIC for OS and PFS compared to ISS suggest that the limitation in sample size has minimal effect and validates this model.

In conclusion, our findings add to previous studies and confirm that sFLC, and particularly the sFLCR, has a major role in survival of patients with MM. A cut-off of sFLCR≥47 had prognostic value at diagnosis independently of the ISS. The sFLCR can separate myeloma patients into various risk groups with different outcomes to identify high-risk patients who could benefit from a more aggressive treatment and more comprehensive monitoring of the treatment. According to our results, sFLCR should be included in the ISS or a new risk stratification model for patients with newly diagnosed MM. Furthermore, the presence of an altered sFLC ratio at CR represents the existence of a persistent clonal population that is secreting very small amounts of monoclonal protein. Our results indicate that sCR represents a deeper response state compared with conventional CR. Analysis of sFLC ratio was able to identify favourable group of patients and support the inclusion of sFLC ratio as part of the response criteria for MM. Further studies with larger series of patients must be done to confirm the prognostic value of sFLC at diagnosis and to validate the response state of sCR.
